# Molecular detection of extended-spectrum β-lactamase-producing *Klebsiella pneumoniae* isolates of chicken origin from East Java, Indonesia

**DOI:** 10.14202/vetworld.2019.578-583

**Published:** 2019-04-19

**Authors:** Meutia Hayati, Agustin Indrawati, Ni Luh Putu Ika Mayasari, Istiyaningsih Istiyaningsih, Neneng Atikah

**Affiliations:** 1 Division of Medical Microbiology, Faculty of Veterinary Medicine, Bogor Agricultural University-West Java, Indonesia; 2 Division of Bacteriology, National Veterinary Drug Assay Laboratory, Gunungsindur Bogor-West Java, Indonesia

**Keywords:** antibiotic resistance, chicken, extended-spectrum β-lactamase, *Klebsiella pneumoniae*

## Abstract

**Background and Aim:**

*Klebsiella pneumonia*e is one of the respiratory disease agents in human and chicken. This bacterium is treated by antibiotic, but this treatment may trigger antibiotic resistance. Resistance gene in *K. pneumoniae* may be transferred to other bacteria. One of the known resistance genes is extended-spectrum β-lactamase (ESBL). This research aimed to study *K. pneumoniae* isolated from chicken farms in East Java, Indonesia, by observing the antibiotic resistance pattern and detect the presence of ESBL coding gene within the isolates.

**Materials and Methods:**

A total of 11 *K. pneumoniae* isolates were collected from 141 chicken cloacal swabs from two regencies in East Java. All isolates were identified using the polymerase chain reaction method. Antimicrobial susceptibility was determined by agar dilution method on identified isolates, which then processed for molecular characterization to detect ESBL coding gene within the *K. pneumoniae* isolates found.

**Results:**

The result of antibiotic sensitivity test in 11 isolates showed highest antibiotic resistance level toward ampicillin, amoxicillin, and oxytetracycline (100%, 100%, and 90.9%) and still sensitive to gentamicin. Resistance against colistin, doxycycline, ciprofloxacin, and enrofloxacin is varied by 90.9%, 54.5%, 27.3%, and 18.2%, respectively. All isolates of *K. pneumoniae* were classified as multidrug resistance (MDR) bacteria. Resistance gene analysis revealed the isolates harbored as *bla*_SHV_ (9.1%), *bla*_TEM_ (100%), and *bla*_CTX-M_ (90.9%).

**Conclusion:**

All the bacterial isolates were classified as MDR bacteria and harbored two of the transmissible ESBL genes. The presence of antibiotic resistance genes in bacteria has the potential to spread its resistance properties.

## Introduction

*Klebsiella pneumoniae* is a Gram-negative, rod-shaped, non-motile, encapsulated opportunistic pathogen. This bacterium is part of the*Enterobacteriaceae* family, anaerobic facultative, measuring 1.0 mm with 0.6-6.0 mm length with mucoid colony [1]. *K. pneumoniae* causes an abscess in the liver and included as one of the emerging diseases in the world. Between the mid-80s and 90s, there was an increasing report of liver abscess or metastatic infection in eyes caused by hypervirulent *K. pneumoniae* in Taiwan, Hong Kong, and Singapore. This increase is also found in North America, South America, the Caribbean, Europe, Middle East, Australia, Africa, and South Africa [2]. *K. pneumoniae* is also responsible for a significant proportion of hospital-acquired infections including septicemias, urinary tract infections, pneumonia, and soft tissue infections, especially in the immunocompromised hosts such as the neonate [3]. Other than nosocomial infection, *K. pneumoniae* also spread through foodstuff and thus often regarded as a foodborne disease agent. *K. pneumoniae* can be found in foodstuffs such as seafood, frozen food, and fresh chicken meat [4]. *K. pneumoniae* is not only a pathogen to human, but it is also a respiratory disease agent in chicken which can cause production loss or even death. In research conducted by Alexandria University, Egypt, 10% of 150 specimens taken from chicken with respiratory disease contained *K. pneumoniae* [5].

Antibiotics are commonly used as therapy and disease control in humans and animals. However, widespread use of antibiotic may also trigger the rise of antibiotic resistance. Cases of multidrug resistance (MDR) in *K. pneumoniae* have been repeatedly reported. MDR is a case of isolates resistant against more than three types of antibiotics. MDR cases, especially from extended-spectrum β-lactamase (ESBL) group, have been reported from *Klebsiella* spp. isolate test in Shandong Poultry Slaughterhouse, China, where 96.7% of bacteria isolates were resistant against more than three types of antibiotics. Antibiotics tested were ceftazidime, cefoperazone, cefotaxime, cefepime, ampicillin, kanamycin, chloramphenicol, tetracycline, and ciprofloxacin [6]. A similar trend of resistance was observed among 102 *K. pneumoniae* from 17 free-range chicken samples in Cape Town, South Africa. The isolates exhibited a high level of resistance toward ampicillin (66.7%), nalidixic acid (61.8%), tetracycline (59.8%), and trimethoprim (50.0%) but highly susceptible toward gentamicin (3.9%) and ciprofloxacin (4.8%). Almost 40% of the isolates were found to be MDR *K. pneumoniae* strains [1]. Another study, MDR enteric bacteria were isolated from turkey, cattle, and chicken farms and retail meat products in Oklahoma. Among the isolated species, multidrug-resistant *K. pneumoniae* was isolated from most of the collected samples. Therefore, a total of 132 isolates of *K. pneumoniae* were characterized. All isolates were resistant to ampicillin, tetracycline, streptomycin, gentamycin, and kanamycin [7]. This often causes antibiotic treatment to be ineffective and requires repeated antibiotic treatment using several different antibiotic groups.

Resistance gene in *K. pneumoniae* may be transferred to other bacteria. Based on several researches, it is known that resistance gene from *K. pneumoniae* can be transferred *in vitro* and *in vivo* [8,9]. One of the resistance genes located in the plasmid is ESBL coding gene. ESBL is an enzyme produced by Gram-negative bacillus bacteria that can hydrolyze penicillin, cephalosporin (Groups 1, 2, and 3), and monobactam [10]. ESBL coding genes and other antibiotic resistance genes which are part of antibiotic resistance integrons (ARI) are transmissible horizontally through plasmid sequence insertion, transposon and conjugation. Several reports stated that the presence of integrons in ESBL coding gene plasmid has a high correlation with MDR. This may occur if the ARI and ESBL coding genes are within one integron complex or within one plasmid [11,12].

Majority of a poultry farm in Indonesia uses antibiotics as treatment (86.7%), prevention (46.7%), and growth promotor to increase feed efficiency (10%) [13]. This means the risk of antibiotic resistance in Indonesian poultry farm is very high and thus requires further study. This research aimed to study *K. pneumoniae* isolated from chicken farms in East Java, Indonesia, by observing the antibiotic resistance pattern and detect the presence of ESBL coding gene within the isolates.

## Materials and Methods

### Ethical approval

Ethical approval was not required in this study. However, samples were collected as per the standard sample collection procedure.

### Sample collections

A total of 141 cloacal swabs were collected from healthy chickens with sterile swabs. The samples were taken from 47 intensive rearing system farms during April-May 2017 spread in two regencies (Kediri and Blitar) in East Java, Indonesia.

### Isolation and Identification of K. pneumoniae

The cloacal swabs were inserted into test tubes containing sterile brain heart infusion broth (BHIB) (BD, Sparks, MD, USA) and incubated at 37°C for 24 h. Loopfuls of the turbid suspension in the BHIB tubes were aseptically cultured on eosin methylene blue (EMB) agar (BD, Sparks, MD, USA) and incubated at 37°C for 18-24 h. On EMB agar, *Klebsiella* spp. produced large, mucoid, pink-to-purple, and nonmetallic-green sheen colonies [14]. *Klebsiella* spp. was subcultivated on heart infusion agar (HIA) (BD, Sparks, MD, USA) at 37°C for 24 h. Identification was carried out by biochemical tests using Microgen GN-ID A system (Microgen Bioproducts Ltd., United Kingdom).

Confirmatory test of *K. pneumoniae* was conducted according to the polymerase chain reaction (PCR) methods [15]. DNA was isolated using PrepMan reagent (Applied Biosystems, Foster City, CA) according to manufacturer’s instructions. Species-specific primers were used for the amplification of *rpoB* gene of *K. pneumoniae* (F-CAACGGTGTGGTTACTGACG and R-TCTACGAAGTGGCCGTTTTC). PCR was performed using HotStarTaq™ DNA Polymerase kit (Qiagen, Valencia, CA). The PCR master mix reagents for each genes target were as follows: 2.5 µL PCR buffer (10×), 2.5 µL MgCl2 (25 mM), 0.5 µL dNTP mix (at 200 µM), 2.5 µL primary forward (10 µM), 2.5 µL primary reverse (10 µM), 0.125 µL HotstarTaq DNA polymerase, 5 µL DNA template, and dH2O up to 25 µL.

The PCR process was used a thermocycler with a predenaturation cycle of 95°C for 15 min, followed by a DNA amplification stage with 30 cycles (94°C for 1 min, 55°C for 1 min, and 72°C for 1 min) and final extension cycle of 72°C for 10 min. Electrophoresis was conducted using 1.5% agarose gel, Tris-acetate-EDTA (TAE) buffer 1×concentration, SYBR safe, loading dye, and DNA ladder/marker 100 bp.

### Antimicrobial susceptibility testing

Each *K. pneumoniae* isolate was tested for antimicrobial susceptibility using eight antibiotics: Ampicillin, amoxicillin, doxycycline, gentamicin, oxytetracycline, enrofloxacin, colistin and ciprofloxacin (Sigma-Aldrich, St. Louis, MO, USA). Agar dilution was a quantitative susceptibility testing method because minimum inhibitory concentration values can be obtained using the method. In this method, two-fold serial dilutions of an antibiotic made in Mueller-Hinton agar (MHA) (BD, Sparks, MD, USA). The concentration ranges evaluated were 0.25-512 µg/ml. Bacterial suspensions were inoculated on the MHA and incubated at 37°C for 18-20 h. The results were interpreted according to performance standards for antimicrobial susceptibility testing 27^th^ edition, Clinical and Laboratory Standards Institute guidelines. Antimicrobial susceptibility test against colistin was interpreted according to EUCAST guidelines. *Escherichia coli* reference strain ATCC 25922 was used as the susceptible reference strain in all tests [16,17].

### Genomic DNA extraction

Isolates were subcultured at 37°C overnight in HIA (BD, Sparks, MD, USA). The bacteria were extracted by boiling method using Ultra Sample Preparation Reagent (PrepMan^®^, Applied Biosystems, Foster City, CA).

### Characterization of ESBL genes using PCR

The presence of genes encoding ESBL was detected using PCR amplification. This study detected *bla*_SHV_ [18], *bla*_TEM_ [6], and *bla*_CTX-M_ [19] gene using primers from the previous study. The PCR master mix reagents for each genes target were as follows: 2.5 µL PCR buffer (10×), 2.5 µL MgCl2 (25 mM), 0.5 µL dNTP mix (at 200 µM), 2.5 µL primary forward (10 µM), 2.5 µL primary reverse (10 µM), 0.125 µL HotstarTaq DNA polymerase, 5 µL DNA template, and dH_2_O up to 25 µL.

The PCR process was used a thermocycler with a predenaturation cycle of 95°C for 15 min, followed by a DNA amplification stage with 30 cycles (94°C for 1 min, 54.5-60°C for 1 min, and 72°C for 1 min) and final extension cycle of 72°C for 10 min. Electrophoresis was conducted using 1.5% agarose gel, TAE-EDTA buffer 1×concentration, SYBR safe, loading dye, and DNA ladder/marker 100 bp. A positive sample would show DNA band on the appropriate amplicon length as shown in Table-1 [6,18,19]. *K. pneumoniae* ATCC 700603 was used as a positive control against gene *bla*_SHV_.

**Table-1:** Primers sets for detection of ESBL encoding genes from *Klebsiella pneumoniae*.

No.	Resistance gene	Primer	Annealing Temperature	Amplicon (bp)	References
1	*bla*_SHV_	(F) 5′- CCTGTTAGCCACCCTGCC-3′	60°C	768	[18]
		(R) 5′- CCGCAGATAAATCACCAC-3′			
2	*bla*_TEM_	(F) 5′-ATTTCCGTGTCGCCCTTAT-3′	54.5°C	759	[6]
		(R) 5′-CTACGATACGGGAGGGCTTA-3′			
3	*bla*_CTX-M_	(F) 5-′ATGATGAAAAAATCGTTATGC-3′	57°C	489	[19]
		(R) 5′-CAGCATCTCCCAGCCTAAT-3′			

ESBL=Extended-spectrum b-lactamase

## Results

Twenty-three isolates suspected as *Klebsiella* spp. were collected from 141 chicken cloacal swabs. From biochemical analysis obtained, about 13 samples characterize as the *K. pneumoniae*. All of the isolates were tested using PCR method to identify *K. pneumoniae* using gene *rpoB* as a target. The visualization result of isolate PCR product from Blitar and Kediri showed that two isolates were negative to the *rpoB* gene. Hence, it could be confirmed that genotypically there were only 11 positive samples of *K. pneumoniae* (seven isolates from Blitar and four isolates from Kediri Regency).

Antimicrobial susceptibility test in 11 isolates of *K. pneumoniae* showed that all isolates were resistant to three or more antibiotics. The pattern of antimicrobial resistance is shown in Table-2. The highest number of resistances was against ampicillin, amoxicillin, and oxytetracycline (100%, 100%, and 90.9%, respectively) which were the first antibiotics used. Isolates were still sensitive to gentamicin. Susceptibility test of colistin, doxycycline, ciprofloxacin, and enrofloxacin was varied at 90.9%, 45.5%, 45.4%, and 36.4%, respectively. The results of antibiotic resistance are shown in Table-3.

**Table-2:** Antimicrobial resistance pattern of *Klebsiella pneumoniae*.

No.	Antibiotics	Sample code										

		B2c	B12c	B13c	B27a	B29a	B29b	B31b	K3b	K6b	K10b	K15a
1	Ampicillin	R	R	R	R	R	R	R	R	R	R	R
2	Amoxicillin	R	R	R	R	R	R	R	R	R	R	R
3	Oxytetracycline	S	R	R	R	R	R	R	R	R	R	R
4	Doxycycline	R	R	R	R	R	R	S	S	S	S	S
5	Ciprofloxacin	S	R	R	I	I	I	R	S	S	S	S
6	Enrofloxacin	I	R	R	I	I	I	I	S	S	S	S
7	Colistin	S	S	S	R	S	S	S	S	S	S	S
8	Gentamicin	S	S	S	S	S	S	S	S	S	S	S
Total		3	6	6	4	4	4	4	3	3	3	3

R=Resistance, S=Susceptible, I=Intermediate

**Table-3:** Antimicrobial susceptibility rate of *Klebsiella pneumoniae* isolates.

Group	Antibiotic	Resistance (%)	Intermediate (%)	Susceptible (%)
b-lactam	Ampicillin	100	0	0
	Amoxicillin	100	0	0
Tetracycline	Oxytetracycline	90.9	0	9.1
	Doxycycline	54.5	0	45.5
Fluoroquinolone	Ciprofloxacin	27.3	27.3	45.4
	Enrofloxacin	18.2	45.4	36.4
Polypeptide	Colistin	9.1	-	90.9
Aminoglycoside	Gentamicin	0	0	100

**Figure-1:**
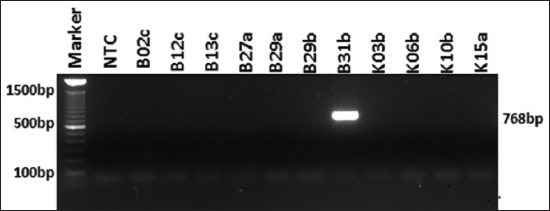
Amplification of *bla*_SHV_ gene on *Klebsiella pneumoniae*. A total of one isolate (B31b) showed a positive result of *bla*_SHV_. NTC: Non template control.

**Figure-2:**
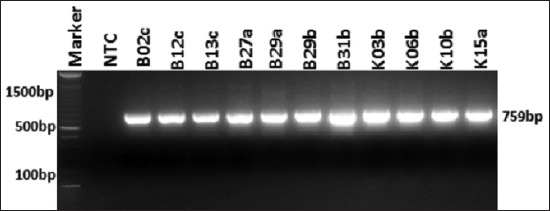
Amplification of *bla*_TEM_ gene on *Klebsiella pneumoniae*. All isolates showed positive results of *bla*_TEM_. NTC: Non template control.

**Figure-3:**
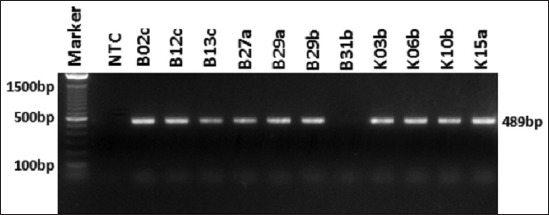
Amplification of *bla*_CTX-M_ gene on *Klebsiella pneumoniae*. A total of 10 isolates showed positive results of *bla*_CTX-M_. NTC: Non template control.

Molecular detection of ESBL encoding genes was performed on all isolates of *K. pneumoniae* resistant against β-lactam antibiotics. From 11 isolates of *K. pneumoniae*, the *bla*_SHV_ gene was only detected in one sample (0.9%) isolates (Figure-1). The *bla*_TEM_ gene was detected in all *K. pneumoniae* isolates (100%) (Figure-2). o Based on PCR detection of the *bl*a_CTX-M_ gene, we found one isolate was negative while ten other isolates were positive for *bla*_CTX-M_ (90.9%) (Figure-3). The presence of these three ESBL encoding genes indicated that *K. pneumoniae* produces ESBL.

## Discussion

The PCR results of *rpoB* gene showed from 13 suspected isolates in the biochemical analysis; only 11 samples showed a DNA band at a 108 bp amplicon length. These different results could be due to the false-positive results in biochemical analysis due to a delayed positivity test in ornithine decarboxylase and motility tests. This test will give a negative result to *K. pneumoniae* but for *Enterobacter* spp. and *Shigella* spp. will provide positive results. This biased test result could be avoided by increasing the incubation time from 24 h to 2-5 days [20]. The use of genotyping techniques may address the problem of phenotypic identification. Identification of *K. pneumoniae* using *rpoB* gene target could be used more accurate and faster test times. Rapid and accurate identification would make it easier to take the most appropriate treatment [21].

The result of antimicrobial susceptibility tests shows that all *K. pneumoniae* isolates are the MDR bacteria. The case of MDR on *K. pneumoniae* causes longer treatment and difficult to cure. The antibiotic resistance pattern was expected to be a guide in choosing the right antibiotics for treatment. Widespread use of antibiotics without strict supervision may trigger antibiotics resistance. Until now, antibiotic is easy to obtain and may be applied without veterinarian supervision. As many as, 72.3% of chicken farmers in Indonesia are still using antibiotics without veterinarian supervision [13].

All the samples of cloacal swabs were taken from intensive farm. Intensive broiler rearing systems are associated with greater use of antibiotics for therapeutic purposes due to high stocking densities, stressful conditions, and fast growth rates. FAO (2008) reported that irrespective of dose, an estimated 75% of antimicrobial agents administered to intensively reared broilers may be excreted into the environment, leading to the development of antimicrobial-resistant bacterial strains in humans. Furthermore, evidence indicates that antimicrobial residues in manure might also be responsible for the contamination of soil, surface water, and groundwater resources close to farms involved in intensive broilers rearing activities [22].

Tested β-lactam groups were ampicillin and amoxicillin. Antimicrobial susceptibility test showed that all isolates were resistant to ampicillin and amoxicillin. This antibiotic has a broad-spectrum activity and thus often used for many applications. Since its introduction, the usage of ampicillin and amoxicillin in farms is very high. They are used as individual and flock treatment and as antibiotic growth promotor given from feed or drinking water.

From the test results, resistance against oxytetracycline is 90.9%. This high resistance induces the use of second generation of tetracycline, namely doxycycline. However, resistance to doxycycline also rises, which can be seen from the percentage of resistant isolates, 54.5%. Tetracycline is an antibiotic commonly used in farms in Indonesia and worldwide dueto its efficacy as a broad-spectrum antibiotic, easily absorbed, cheap, and has minimum side effects [13,23].

The most widely used antibiotics in chicken farms other than oxytetracycline are enrofloxacin and ciprofloxacin. Based on susceptibility test of ciprofloxacin and enrofloxacin from 11 isolates of *K. pneumonia*e, we found 45.4% were susceptible to ciprofloxacin and 36.4 % isolates were susceptible to enrofloxacin. Fluoroquinolone has been classified by the World Health Organization as critically important in human medicine for its ability to treat infections from *Campylobacter* spp., *Salmonella* spp., and *E. coli* infections. To prevent further resistance, treatment using fluoroquinolone has been limited only for individual treatment, not a group. Overuse in farm-animal species can contribute to higher levels of resistance in human *Salmonella, E. coli*, and *Campylobacter* infections. Those countries which have banned or have never used fluoroquinolones in poultry have much lower levels of resistance in human bacterial infections than those countries which continue to use the drugs in poultry [24].

Gentamicin is still sensitive to all *K. pneumoniae* isolates. Gentamicin is aminoglycoside group highly important in treating bacterial infection in livestock and pet. According to OIE, these antibiotics were included in veterinary critically important, and if it ever loses its effectivity, there is a need for other antibiotics to replace. Its alternative is fluoroquinolone and colistin; however, these two antibiotics have been limited for usage in animal, being included as critically important in human medicine [25]. One of the *K. pneumoniae* isolates is already resistant to colistin.

ESBL bacteria can be identified by detecting the presence of ESBL encoding genes [26]. This research results showed that *bla*_TEM_ gene was found at 100% isolates and followed by *bla*_CTX-M_ (90.9%). The *bla*_SHV_ gene is only found in one isolate from 11 *K. pneumoniae* isolates. Other studies of *K. pneumoniae* have been widely practiced. Some investigation showed that the dominant genotype found was the *bla*_CTX-M_ gene [27,28]. The ESBL type was often seen as single or in combination. In this study, ESBL encoding genes were detected in all *K. pneumoniae* isolates from chicken.

Mobile genetic elements such as transposons, insertion sequences, and integrons in bacteria cause the ESBL gene to move quickly from animal to human or vice versa. The genetic factors could also spread the nature of resistance to other bacteria in the animal’s gastrointestinal tract. The bacteria then spread from farm to surrounding environment through waste facilitated by bad hygiene and sanitation, which contaminate soil and water around farm. ESBL bacteria were also detected in vegetables, soil, and water around farm and market [28-30]. Contamination in chicken product and other foodstuff may happen if processing is not done with good hygiene [4,6]. This shows that ESBL bacteria not only cause nosocomial infection but also cause community infection and foodborne disease.

From the results of antimicrobial susceptibility test, it was known that all isolates of *K. pneumoniae*, also, having an ESBL gene were also an MDR bacteria. The presence of MDR-ESBL bacteria is a threat to public health and livestock. These conditions could result in limited treatment options. Furthermore, MDR-ESBL bacteria triggered the use of antibiotic that has no longer been used for toxicity, such as colistin [31]. Measures that could be undertaken were building surveillance programs, conducting surveillance on feed and livestock. Breeders also need to improve biosecurity practice. Litter and manure waste must be properly managed in intensive production systems, to prevent the contamination of air, soil, and water, as well as negative consequences for human health [32].

## Conclusion

Eleven *K. pneumoniae* isolates could be isolated from chicken cloacal swabs in the chicken farms in East Java. All *K. pneumoniae* were classified as MDR bacteria. Through PCR testing, ESBL encoding genes could be identified in all isolates. The presence of resistant encoding gene in bacteria has the potential to spread its resistance to the other bacteria in the gastrointestinal tract of chickens as well as in the livestock environment.

## Authors’ Contributions

MH designed the study and drafted the manuscript under the supervision of AI and NLPIM. NA and II collected samples and compiled the resource materials. MH and NA performed the test and data analysis under the supervision of II. MH conducted molecular detection of resistant gene by PCR under the supervision of AI and NLPIM. All authors have read and approved the final manuscript.
